# Cancer Stem Cells and Their Microenvironment: Biology and Therapeutic Implications

**DOI:** 10.1155/2017/3714190

**Published:** 2017-02-26

**Authors:** Eunice Yuen-Ting Lau, Nicole Pui-Yu Ho, Terence Kin-Wah Lee

**Affiliations:** ^1^Department of Applied Biology and Chemical Technology, The Hong Kong Polytechnic University, Kowloon, Hong Kong; ^2^State Key Laboratory for Liver Research, The University of Hong Kong, Pokfulam, Hong Kong

## Abstract

Tumor consists of heterogeneous cancer cells including cancer stem cells (CSCs) that can terminally differentiate into tumor bulk. Normal stem cells in normal organs regulate self-renewal within a stem cell niche. Likewise, accumulating evidence has also suggested that CSCs are maintained extrinsically within the tumor microenvironment, which includes both cellular and physical factors. Here, we review the significance of stromal cells, immune cells, extracellular matrix, tumor stiffness, and hypoxia in regulation of CSC plasticity and therapeutic resistance. With a better understanding of how CSC interacts with its niche, we are able to identify potential therapeutic targets for the development of more effective treatments against cancer.

## 1. Introduction

Cancer exists as a heterogeneous population, with different cancer cells showing distinct phenotypic and functional properties, leading to the limitation of therapeutic efficacy and treatment outcomes. In fact, the discovery of the “Cancer Stem Cell (CSC)/Tumor-Initiating Cell (T-IC)” theory provides an alternative explanation for the failure of existing therapies. Although the idea of CSCs was proposed over a decade ago, the existence of CSCs has been identified in various types of cancer by taking the advantage of available cell surface markers in the last 10 years. In this model, cancer cells are organized in a hierarchy with cancer stem cells (CSCs)/Tumor-Initiating Cell (T-IC) located at the apex [1]. The new concept of CSCs is based on the idea that stem cells are present in cancer tissue, like in normal tissues, and are part of the hierarchy of cells. In other words, just as there are normal stem cells in normal tissues, CSCs are found in tumor tissues. Although the origin of CSCs remains controversial, there is increasing evidence to support that CSCs arise by either mutation from normal stem/progenitor cells or deregulation of genetic programs regulating these cells. These acquired mutations allow normal stem cells to transform from their quiescent and tightly regulated phenotype to constitutively activated ones. This model proposes that CSCs, which share some similar functional properties with normal stem cells, possess the ability to self-renew and initiate tumor formation and generate additional differentiated progenies that compose the heterogeneous tumor bulk. Furthermore, mounting evidence has shown that CSCs are protected by multiple resistance mechanisms, leading to tumor metastasis, therapeutic resistance, and recurrence. Therefore, CSC-targeting therapies represent a promising strategy for the long-term cure of the disease.

And in theory, stem/progenitor cells represent the natural target of tumorigenic mutations since they are possibly the only cells that have the longevity and are endowed with the appropriate capabilities to accumulate the required number of mutations needed to disrupt intrinsic mechanism regulating normal cell proliferation and differentiation [2, 3].

**Figure 1:**
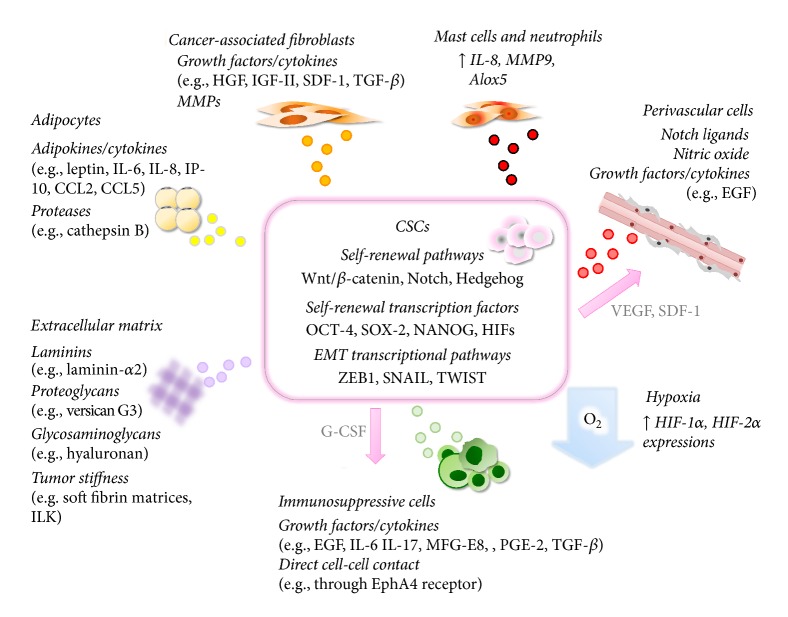
The cross talks between CSCs and their niches. CSCs reside in their habitats, which are specific microenvironments within the tumor consisting of CAFs, masts cells, neutrophils, perivascular cells, adipocytes, ECM, and immune-suppressive cells, as well as hypoxia. By providing various paracrine factors or via direct cell-cell contact, these niches play a crucial role in maintaining CSC plasticity by regulating pathways or transcription factors involved in self-renewal or EMT process. Reciprocally, CSCs can actively recruit some of these niche components to create a microenvironment that is favorable for its survival. For example, CSCs can secrete VEGF and SDF-1 to recruit perivascular cells or produce G-CSF to recruit MDSCs into the tumor microenvironment.

In a normal organ, stem cells reside in a “stem cell niche,” a specific microenvironment that plays a key role in regulating stem cell maintenance and self-renewal by secreting various paracrine factors or by direct cell-cell contact that interferes with self-renewal and differentiation pathways. A similar concept applies to CSCs in which a cancer-specific “cancer stem cell niche” is also present and interactions with this niche are essential for maintaining the CSC population. Tumor specific microenvironments comprise stromal cells, immune cells, networks of cytokines and growth factors, hypoxic regions, and the extracellular matrix (ECM) (Figure 1). These environmental factors collectively maintain the stemness of CSCs through altering self-renewal pathways, such as the Wnt/*β*-catenin, Notch, and Hedgehog pathways, or by interrupting the master transcriptional regulators that sustain embryonic stem cell self-renewal, such as NANOG, OCT-4, and SOX-2 [2, 3]. Furthermore, extensive evidence has revealed that cancers do not strictly follow the CSC model and the actual CSC model is more complex and flexible. Given a specific environmental stimulus, certain cancer cells exhibit plasticity, enabling these cells to reversibly convert from differentiated to a stem-like state through dedifferentiation processes, such as the epithelial-to-mesenchymal transition (EMT) [4]. Considering the essential role of the tumor microenvironment in regulating the CSC phenotype, this review will focus on the recent findings on the molecular mechanisms involved in the cross talk between CSCs and their niches which contributes to maintaining the CSC population.

## 2. Stromal Cells

### 2.1. Cancer-Associated Fibroblasts

Cancer-associated fibroblasts (CAFs) are the major components of the tumor stroma [10, 11]. Recent studies have revealed that CAFs are a heterogeneous population, most of which acquire the activated phenotype with increased contractile force, proliferative activity, and enhanced secretion of ECM, proteases, and growth factors. CAFs emerge from multiple origins that widely vary among different cancer types. Several studies have shown that cancer cells could actually secrete signaling molecules, such as basic fibroblast growth factor (bFGF), transforming growth factor beta (TGF-*β*), platelet-derived growth factor (PDGF), and interleukin- (IL-) 6 to “educate” resting fibroblasts to become CAFs [12–15], and in turn, CAFs promote tumor growth and sustain the stemness property of CSCs in a paracrine manner. Through the secretion of hepatocyte growth factor (HGF), CAFs from colon cancer were demonstrated to support CSC properties through the induction of Wnt/*β*-catenin signaling [16]. More interestingly, the paracrine activation of Wnt/*β*-catenin signaling by CAFs could restore the stem-like features of non-CSCs, thereby expanding the pool of these cells. Using conditioned media from CAFs, we showed that CAFs from liver cancer promote cancer stemness through the noncanonical induction of the Notch signaling effector HEY-1 mediated by HGF [17]. A recent study also demonstrated that CAFs in lung cancer induce the expression of the NANOG transcription network through paracrine insulin-like growth factor II (IGF-II)/IGF-1R signaling [18]. EMT is the process where cancer cells acquire a mesenchymal trait and become more invasive and metastatic. Cancer cells that have undergone EMT typically acquire an increased stemness property because some of the EMT-mediating transcription factors, such as Snail and ZEB1, are essential for self-renewal. Several studies have also shown that the activation of EMT could induce the generation of the CSC population [19, 20]. In prostate cancer, CAFs can elicit EMT and increase the stemness properties of cancer cells through the secretion of MMPs [13]. Furthermore, CAFs from breast cancer have been reported to promote the EMT of cancer cells via the secretion of stromal-derived factor 1 (SDF-1) and TGF-*β*1 [21, 22], providing additional support, suggesting that CAFs play a crucial role in promoting cancer stemness.

### 2.2. Adipocytes

Obesity is a well-recognized risk factor of several common human malignancies, including breast cancer, colon cancer, and liver cancer [23]. In addition to its epidemic significance, emerging studies have uncovered the functional role of adipose tissues in carcinogenesis and cancer progression, particularly in cancers with adipose tissue constituting a major part of the tumor microenvironment. Adipose tissue primarily comprises adipocytes and a variety of cells that make up the stromal vascular fraction. In addition to its lipid storage function, adipocytes can actively secrete multiple adipokines and cytokines, such as leptin, adiponectin, IL-6, MCP-1, and TNF-*α*, during excessive adiposity [24]. In addition to its role in lipid homeostasis, many of these adipokines and cytokines are proinflammatory, which attract the infiltration of inflammatory cells, particularly macrophages, causing chronic inflammation to promote cancer growth and metastasis. Furthermore, some of these adipocyte-secreted adipokines/cytokines were directly involved in regulating CSCs. In breast cancer, the expression of leptin receptor is highly upregulated in tumor tissue, particularly in the CSC subpopulation, as driven by the self-renewal associated transcription factors OCT-4 and SOX-2. The secretion of leptin by adipocytes activates the STAT3 signaling in CSCs and induces the expression of OCT-4 and SOX-2, in turn stimulating the expression of leptin receptor, which maintains a self-reinforcing signaling cascade to expand the CSC population and promote tumor growth [25]. Another study showed that the coculture of adipocytes and breast cancer cells stimulates the production of various cytokines that promote cancer stemness through the Src/SOX-2/miR-302b signaling pathway [26]. In prostate cancer, where obesity is associated with a more aggressive phenotype, adipocytes produce cathepsin B (CTSB) upon coculture with prostate cancer cells to support the self-renewal of CSCs [27]. Adipocytes from colorectal cancer are also demonstrated to enhance cancer stemness, and their oncogenic function can be impaired by grape seed extract, a well proven agent with anticolorectal cancer activity, through inducing the “browning” of adipocytes [28].

### 2.3. Perivascular Cells

Angiogenesis is essential for tumor growth and metastasis. With the excessive production of proangiogenic factors by cancer cells, tumors typically develop disorganized and rich blood vessel networks to meet the high demand on oxygen and nutrients required for tumor outgrowth. CSCs promote tumor angiogenesis. For example, in brain, skin, pancreatic, and liver cancer, the CD133^+^ CSC populations produce higher levels of proangiogenic factors, such as vascular endothelial growth factor (VEGF) and SDF-1, recruit more endothelial cells, and stimulate more tube formation compared with their differentiated CD133^−^ counterparts [29–31]. Intriguingly, glioblastoma stem cells, which reside in the perivascular niche, undergo differentiation to generate vascular pericytes and endothelial cells to expand tumor vascularization [32, 33]. Indeed, a mean of approximately 60% of endothelial cells in glioblastoma are derived from neoplastic cells [33]. In turn, CSCs reside in close proximity to the perivascular niche, which provides functional support. Strong evidence suggests that vascular endothelial cells play a key role in maintaining CSCs. In the context of glioblastoma, endothelial cells provide Notch ligands to neighboring CSCs, activating Notch signaling and promoting CSCs self-renewal [34]. In another study, perivascular endothelial cells were demonstrated to activate Notch signaling in glioma stem cells through another soluble factor, nitric oxide [35]. A similar observation was also made in colon cancer, suggesting that endothelial cells secrete the Notch ligand Jagged-1 to promote colon CSC phenotype [36]. A recent study on head and neck cancer also highlighted a role for endothelial cells in regulating CSCs, in which endothelial cells were shown to secrete epidermal growth factor (EGF) to induce EMT and promote cancer stemness [37]. Together, these findings reveal an intriguing reciprocal interaction between CSCs and perivascular cells.

### 2.4. CSCs and Immune Evasion

Tumor immune escape is a fundamental step for tumor development and the major reason for the failure in cancer immunotherapy. Cancer cells evade the infiltration and the cytotoxic function of natural killer (NK) T cells and CD8^+^ cytotoxic T cells through various strategies, including the active attraction of immune-suppressive cells, production of immune-suppressive factors, and the activation of “immune checkpoints” that induce anergy or apoptosis in T lymphocytes to downmodulate immune functions [38, 39]. Several studies have revealed that the activation of prosurvival pathways, such as PI3K/AKT, in CSCs not only facilitates escape from conventional chemotherapies but also confers immune evasion [40]. The expression of MHC-I and MHC-II proteins, required for recognition by T lymphocytes to elicit immune responses, is also downregulated in CSCs [41]. In head and neck cancer, the programmed death-ligand 1 (PD-L1), which binds to the programmed death 1 (PD-1) receptor on T cells to suppress its function, is selectively expressed on CD44^+^ CSCs [42]. Furthermore, it has been well documented that CSCs actively recruit immune-suppressive cells into the tumor microenvironment. In addition to functions in modulating immune cells, these tumor-associated immune-suppressive cells, which mainly include tumor-associated macrophages, myeloid-derived suppressor cells (MDSCs), T-regulatory (Treg) cells, and NK cells, have been widely demonstrated to support CSCs through multiple pathways.

### 2.5. Tumor-Associated Macrophages

Macrophages are classified into M1- and M2-polarized subtypes. The M1-subtype secretes inflammatory cytokines and reactive oxygen intermediates and presents antigen to tumor suppressive T cells. However, the M2-subtypes, which are tumor promoting, induce T cell anergy, produce extracellular matrix components, repair damaged tissues, and induce angiogenesis [43–45]. Although the origins of macrophages in many cancers remain uncertain, most of the macrophages recruited to the tumor microenvironment, known as the TAMs, become the tumor supportive M2 subtype [46]. In glioblastoma, glioma CSCs activate the STAT3 pathway to produce cytokines, which recruit and polarize macrophages to become M2-like [47]. After recruitment, TAMs, in turn, serve as a CSC niche to support CSC growth. For example, in breast cancer, the physical interaction between TAMs and CSCs activates the EphA4 receptor on CSCs and the downstream Src and NF-*κ*B pathways, which promote self-renewal [48]. In a murine model of breast cancer, TAMs are also demonstrated to promote CSC phenotypes in breast cancer cells through the EGF-mediated STAT3/SOX-2 cascade, and this cross talk could be abrogated by small molecule inhibitors against EGFR or STAT3 [49]. TGF-*β*1 and IL-6 are predominantly produced by TAMs in hepatocellular carcinoma (HCC), which induce EMT and activate the STAT3 pathway, respectively, to promote liver CSC properties [50, 51]. Milk-fat globule EGF-8 (MFG-E8), a growth factor identified to involve in phagocytosis and immune suppression [52, 53], is secreted by TAMs to activate STAT3 and Hedgehog pathways that trigger tumorigenicity and drug resistance in CSCs from various cancers [54]. It is clear that the interplay between CSCs and TAMs coordinately regulates tumor progression.

### 2.6. Myeloid-Derived Suppressor Cells

MDSCs are a heterogeneous population of myeloid-originated progenitor cells. In mice, these cells are characterized as CD11b^+^Gr1^+^, whereas in humans, their phenotype is Lin^−^HLA^−^DR^−^CD33^+^ or CD11b^+^CD14^−^CD33^+^ [55–58]. As the name indicates, the main feature of MDSCs is their function on immunosuppression. MDSCs suppress immune function primarily through multiple mechanisms, including the production of arginase, inducible nitric oxide synthase (iNOS), reactive oxygen species (ROS), cyclooxygenase-2 (COX-2), and TGF-*β*, which together inhibit the proliferation and function of T cells [59, 60]. Recent studies have demonstrated that MDSCs are actively recruited into tumors and these tumor-associated MDSCs play an important role in tumor progression. The recruitment of MDSCs into tumor sites is primarily mediated by various cancer cells that produce chemokines, including CCL2, CCL15, CXCL5, and CXCL12 [61–64]. MDSCs are implicated in multiple stages of tumor progression, particularly the regulation of CSCs. In ovarian cancer, coculture with MDSCs stimulates the expression of miR-101 in cancer cells, which regulates CtBP2 to control the expression of stemness genes, such as NANOG, OCT-4, and SOX-2 [65]. In syngeneic mammary tumor models, CSCs displayed the elevated production of granulocyte colony-stimulating factor (G-CSF), which stimulates the recruitment of MDSCs into the tumor microenvironment. MDSCs reciprocally enhance CSC properties through the activation of Notch signaling [66]. Furthermore, tumor-infiltrated MDSCs, which showed the activation of STAT3 signaling, can enhance the stemness of pancreatic cancer cells through the induction of EMT, with a concomitant increase in the expression of stemness genes, including Snail, Slug, ZEB1, NANOG, and OCT-4 [67].

### 2.7. T-Regulatory Cells

The fine cross talk between CSCs and immunosuppressive cells also involves Treg cells. Treg cells are defined by the CD4^+^CD25^+^FOXP3^+^ T cell subpopulation, with FOXP3 as an important transcriptional regulator of Treg cell development and function [68]. Treg cell-mediated immunosuppression primarily occurs through the production of various cytokines, such as IL-10, IL-35, and TGF-*β*, direct cell-cell contact via gap junctions, or metabolic disruption in which CD39 and CD73, expressed on Treg cells, facilitate the conversion of ATP to adenosine, which suppresses cytotoxic T cell and/or NK cell activity [69–71]. In tumors, Treg cells are accumulated by various mechanisms, primarily involving chemokine attractions. For example, the chemokines CCL22 and CCL28 are produced by tumor cells to attract CCR4- and CCR10-expressing Treg cells, respectively, leading to the accumulation of Treg cells in various human cancers [72–74]. Indeed, the number of Treg cells inside the tumor microenvironment is associated with clinical outcome. The higher number of Treg cells within the tumor is correlated with poor prognosis in a wide array of cancers, including gastric, esophageal, pancreatic, liver, and breast cancers [75–78]. In addition to its immune-suppressive role, the functional importance of tumor-infiltrating Treg cells in regulating CSCs is starting to emerge. A recent report demonstrated that, under hypoxia, FOXP3^+^ Treg cells are induced to express IL-17, which drives the expansion of CSCs through the activation of Akt and MAPK signaling pathways in colorectal cancer, evidenced by the increase in the expression of colorectal CSC markers, including CD133, CD44s, and EpCAM [79]. Furthermore, Treg cells produce and secrete prostaglandin (PGE2) for immunosuppression, and PGE2 has been implicated in the regulation of CSC properties in colorectal cancer through NF-*κ*B [80, 81].

### 2.8. Natural Killer Cells

The ability of natural killer (NK) cells to kill or spare depends on their expression of activating (mostly stress-induced proteins) and inhibitory (in particular MHC class I molecules) ligands on the surface of target cells. Approximately 95% of peripheral blood NK cells are CD56^dim^CD16^+^ which exerts strong cytotoxic activity. The remaining 5% of peripheral blood NK cells are CD56^bright^CD16^−^ and show cytotoxicity through strong cytokine production. CD133^+^ glioblastoma stem cells that are able to express high levels of the activating DNAM-1 ligands PVR and Nectin-2 and low levels of MHC class I molecules have been reported to be poorly recognized and lysed by NK cells [82]. Their cytotoxic activity was revamped following IL-2 or IL-15 activation [82]. Breast cancer CSCs have also been reported to fail to express detectable levels of NK ligands, which is consistent with metastatic spread [83]. In melanoma and GBM, CSCs are highly resistant to NK cells and become susceptible to NK cytotoxicity only following stimulation with IL-2 [82]. However, the preferential resistance of CSC to NK cells is not the rule, as colon CSCs express lower MHC class I and higher levels of NK-activating ligands, including NKp30L and Nkp44L as compared to differentiated cells, which are responsible for the CSC susceptibility to NK cell killing [84]. Another mechanism by which cancer cells may evade from the cytotoxic effect of NK cells is the induction of apoptosis in microenvironmental immune cells through the interaction of CD95 (Apo1/Fas) with its ligand (CD95L). Interestingly, CD95R/L regulates CSC plasticity and its blockade reduces CSC in different tumor cell models, while activation of CD95R/L increases CSC number and is responsible for CSC reduced sensitivity to CD95-mediated apoptosis [85]. Collectively, CSCs are more refractory to the cytotoxic effect of NK cells in a variety of cancer types.

### 2.9. Other Stromal Cells

There is increasing evidence that mast cells (MCs) and their mediators are involved in the remodeling of the tumor microenvironment. Recent evidence has showed that MC regulates stemness of thyroid cancer through IL-8-Akt-Slug pathway [86]. In prostate cancer, MC increased stem/progenitor cell population via altering LncRNA-HOTAIR/PRC2-androgen receptor- (AR-) MMP9 signals [87]. In addition, neutrophils were found to play a crucial role in regulation of CSC populations. Wculek and Malanchi reported that neutrophils induced expansion of breast CSC population marked by CD24^+^CD90^+^, leading to induction of tumor initiation and lung metastasis [88].

## 3. Hypoxia

Hypoxic microenvironments in tumors result from the rapid growth of cancer cells, which exceeds the limit of blood supply [89]. In response to the hypoxia, the hypoxia-related gene expression is driven through the activated hypoxia-inducible factor (HIF) and transcription factors HIF-1*α* and HIF-2*α* that bind to the hypoxia-regulated element (HRE) gene promoters [90–92]. The capacity of HIFs to promote cancer cell stemness has been well documented. Studies have shown that HIFs can increase the expression of stem cell markers in breast cancer [93]. Bae et al. demonstrated that hypoxia can elevate the expression of the stem cell marker SOX2 in prostate cancer cell lines [94]. In addition, the overexpression of HIF-1*α* has been associated with stem cell marker CD44 in bladder cancer [95]. In addition to HIFs, the hypoxia-mediated overexpression of extracellular carbonic anhydrases, CAIV and CAXII, facilitates cancer cell survival and the maintenance of CSC function [96].

Given that CSC is related to metastasis and cancer cell invasion, the contribution of hypoxia to the enhanced CSC migration has been reported in several studies. The upregulation of EMT-related gene expression under hypoxic stress can enhance the invasiveness and the stem-like properties of cancer [89]. Maeda et al. showed that HIF-1*α* is correlated with the EMT and cell migration in CD133^+^ pancreatic CSCs [97]. In addition to cancer cell invasion, hypoxia contributes to drug resistance by maintaining CSCs in a quiescent state to confer resistance to chemotherapeutics that commonly target actively dividing cancer cells [91]. Studies have reported that hypoxia promotes SOX-2-mediated drug resistance in ovarian CSCs via Notch signaling [98]. The downregulation of HIF-1*α* using a lentivirus-mediated approach can increase the chemosensitivity in triple negative breast cancer [99]. These data demonstrated that hypoxia plays an important role in the CSC niche and is substantially involved in the regulation of cancer cell stemness.

## 4. Extracellular Matrix

The extracellular matrix (ECM) is a collection of biochemical molecules, including proteins, glycoproteins, proteoglycans, and polysaccharides, which compose the basement membrane and interstitial matrix. In normal tissue, ECM is tightly regulated during development and primarily accomplished by controlling the expression or activities of ECM enzymes at the transcriptional and translational posttranslational levels [100]. Abnormal ECM dynamics are a hallmark of cancer. For instance, various collagens, including collagen I, collagen II, collagen III, collagen V, and collagen IX, show increased deposition in the process of tumor formation [101]. In addition, many other ECM components and their receptors such as heparan sulfate proteoglycans and CD44 are frequently overexpressed in cancer [102, 103]. As one of the major parts of the CSC niche, ECM provides both structural and biochemical support to the CSC and plays a critical role in cancer progression. ECM receptors enable the CSC to anchor in the niche where the stem cell properties could be maintained [104]. In addition, the ECM binds to various growth factors that interact with CSCs to maintain stem cells in a proliferative state. For example, in glioblastoma, the growth of glioblastoma stem cells can be enhanced by ECM protein laminin-*α*2 [105]. Versican G3, which is overexpressed in breast carcinoma, can inhibit cell differentiation and promote self-renewal, thereby increasing CSC properties [106, 107]. Matrix metalloproteinases that degrade and modify the ECM are upregulated in breast cancer, facilitating the EMT process [108]. Hyaluronan interacts with the cell surface protein CD44, enhancing CSC properties by activating the stem cell marker NANOG [109]. In addition to the stem cell properties, HA-CD44 interactions can also stimulate the overexpression of proteins for multidrug resistance in cancer and CSC [110]. The changes in ECM dynamics may contribute to the disruption of asymmetric stem cell division, leading to CSC overexpansion [111]. When compared with normal tissues, malignant tumors typically are characterized as stiffer due to contraction of collagen in the extracellular matrix by malignant and stromal cells [112]. On single tumor cell level, tumor stiffness was measured by atomic force microscopy mechanical measurement [113]. For in vivo measurement of tumor stiffness, compression and indentation tests were performed on fresh tumor tissues and orthotopic tumors and subcutaneous tumors derived from multiple HCC cell lines [114]. Matrix stiffness in ECM also played crucial role in regulation of CSC plasticity. Tan et al. demonstrated that melanoma CSCs exhibited plasticity in mechanical stiffening, histone 3 lysine residue 9 (H3K9) methylation,* Sox2* expression, and self-renewal. Three-dimensional (3D) soft fibrin matrices promote H3K9 demethylation and increase* Sox2* expression and self-renewal, whereas stiff ones exert opposite effects [115]. More recently, it was found that breast CSC markers are activated synergistically in response to stiff, hypoxic conditions and that ILK is an essential regulator of breast CSCs [116]. The effect of matrix stiffness on CSC marker expression depends on cancer cell's tissue origin [117].

## 5. Conclusions

Mounting evidence suggests that CSCs are the root of cancers and are responsible for metastasis, resistance to conventional therapies, and tumor relapse. The state and survival of CSCs are controlled by various extrinsic factors derived from the microenvironment where the cells reside. As CSCs have to be eradicated to prevent disease relapse or metastasis, targeting the niche factors that regulate CSCs represents an attractive therapeutic strategy for cancer treatment. Considering the encouraging results of several preclinical studies for such therapeutic approaches, targeting the CSC niche is clinically feasible [118] (Table 1). A better understanding of CSC biology and the cross talk with its niche might enable the identification of potential therapeutic targets for the development of more effective anticancer treatments.

## Figures and Tables

**Table 1:** Strategies of targeting the CSC niches for cancer treatment and their respective development status.

Inhibitors/antibodies	Molecular targets	Phases of development	References
*CAFs*			
PT630 (FAP inhibitor)	FAP-*α*	Preclinical	[5]
NK4 (anti-HGF monoclonal antibody)	HGF/MET	Preclinical	[6]
AMG337 (MET kinase inhibitor)	MET	Preclinical	[7]
Rebimastat (MMP inhibitor)	MMPs	Phase II clinical trial	NCT00040755
AMD3100 (CXCR4 antagonist)	SDF-1/CXCR4	Preclinical	[8]
GC1008 (anti-TGF-*β* monoclonal antibody)	TGF-*β*	Phase II clinical trial	NCT01401062

*Tumor vasculatures*			
Sorafenib (tyrosine kinase inhibitor)	VEGFRs, PDGFRs, KIT	FDA-approved	NDA021923
Sunitinib (tyrosine kinase inhibitor)	VEGFRs, PDGFRs, KIT	FDA-approved	NDA021938
MK0752 (*γ*-secretase inhibitor)	*γ*-secretase	Phase I clinical trial	NCT00106145
OMP21M18 (anti-DLL4 monoclonal antibody)	DLL4	Phase I clinical trial	NCT01189968
OMP52M51 (anti-Notch1 monoclonal antibody)	Notch1	Phase I clinical trial	NCT01778439

*TAMs*			
PLX3397 (CSF-1R inhibitor)	CSF-1R	Phase I/II clinical trial	NCT01596751
AMG820 (anti-CSF-1R monoclonal antibody)	CSF-1R	Phase I/II clinical trial	NCT02713529
Zoledronate, clodronate, ibandronate	Deplete macrophages	Phase III clinical trial	NCT00127205
			NCT00009945
852A (TLR7 agonist)	TLR7	Phase II clinical trial	NCT00319748
Imiquimod (TLR7 agonist)	TLR7	Phase II clinical trial	NCT00899574
			NCT00821964

*MDSCs*			
Tadalafil (PDE-5 inhibitors)	PDE-5	Pilot study	NCT00843635
		Phase II clinical trial	NCT00752115
NCX4016 (Nitric oxide-releasing aspirin derivative)	iNOS and arginase	Phase I clinical trial	NCT00331786
		(Prevention purpose)	
L-NAME (arginase inhibitor)	Arginase	Preclinical	[9]
All-trans retinoic acid	Inducing MDSC differentiation	Phase II clinical trial	NCT00617409

*Treg cells*			
MEDI6383 (OX40 agonist)	OX40	Phase I clinical trial	NCT02221960
Ipilimumab (anti-CTLA4 monoclonal antibody)	CTLA4	FDA-approved	BLA125377

*Hypoxia*			
TH-302 (hypoxia-activated prodrug)	Hypoxia	Phase III clinical trial	NCT01746979
AQ4N (hypoxia-activated prodrug)	Hypoxia	Phase I/II clinical trial	NCT00394628

*ECM*			
PEGPH20 (recombinant hyaluronidase)	Hyaluronan	Phase II clinical trial	NCT01839487
		Phase III clinical trial	NCT02715804

## Abbreviations

CAFs: Cancer-associated fibroblasts
CSCs: Cancer stem cells
CTSB: Cathepsin B
COX-2: Cyclooxygenase-2
EGF: Epidermal growth factor
EMT: Epithelial-to-mesenchymal transition
ECM: Extracellular matrix
bFGF: fibroblast growth factor
G-CSF: Granulocyte colony-stimulating factor
HGF: Hepatocyte growth factor
HIF: Hypoxia-inducible factor
HRE: Hypoxia-regulated element
iNOS: Inducible nitric oxide synthase
IGF: Insulin-like growth factor
IL: Interleukin
MCs: Mast cells
MDSC: Myeloid-derived suppressor cell
NK: Natural killer
PDGF: Platelet-derived growth factor
PD-L1: Programmed death-ligand 1
PGE2: Prostaglandin
ROS: Reactive oxygen species
SDF-1: Stromal-derived factor 1
TGF-*β*: Transforming growth factor beta
VEGF: Vascular endothelial growth factor.
